# Supplementary material for: Crosstalk between MSH2–MSH3 and polβ promotes trinucleotide repeat expansion during base excision repair
Source: Nat Commun. 2016 Aug 22;7:12465. doi: 10.1038/ncomms12465 (PMC4996945; doi:10.1038/ncomms12465)
Supplement: Supplementary Information — Supplementary Figures 1-9, Supplementary Table 1, Supplementary Method and Supplementary Reference [file ncomms12465-s1.pdf]

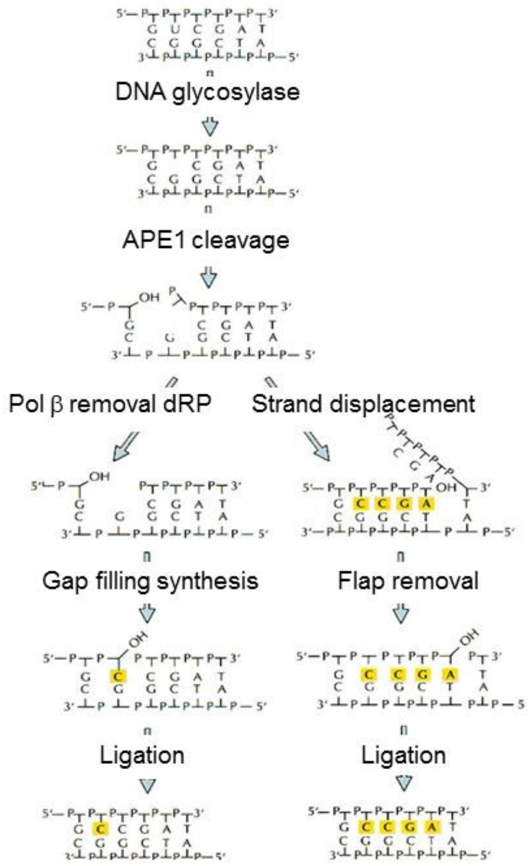

Supplementary Figure 1. A simple schematic view of BER (Taken from *Dianov and Lindahl, Current Biol., 4, 1069-1076, 1994*[1]). A damaged base is removed by a DNA glycosylase, which leaves an abasic site with an unpaired base, a residual deoxyribophosphate (dRP), and a 3'-OH group for polymerase extension. Pol β removes the dRP group either by short patch BER, or it is removed by long patch BER, in which polymerase passage displaces the strand subsequently removed by FEN1. The gap is filled with new bases (yellow), and DNA ligase seals the backbone and reconstitutes duplex DNA.

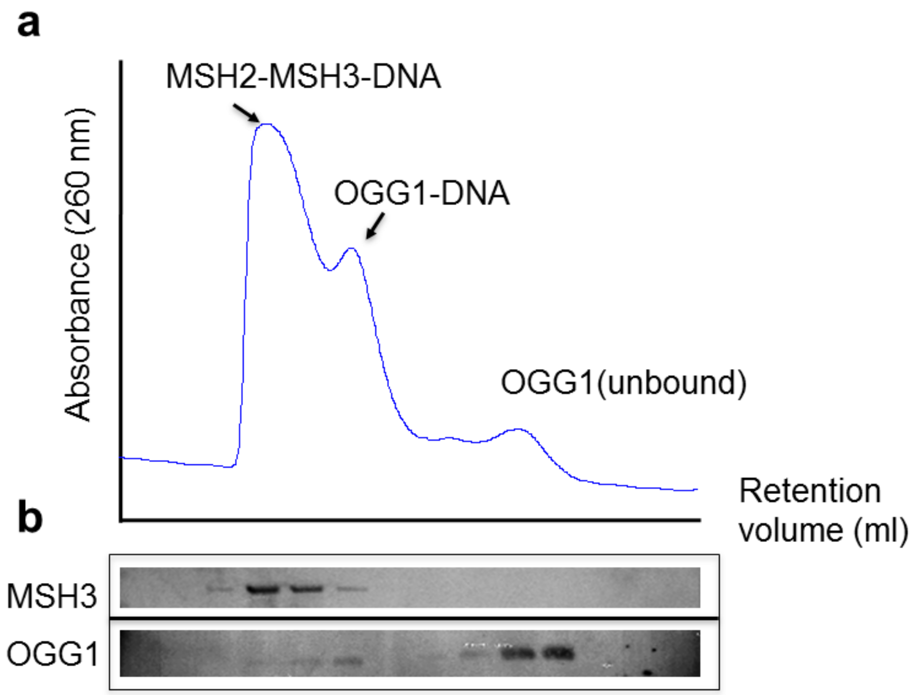

Supplementary Figure 2. There is little interaction between OGG1 and MSH2-MSH3. (**a**) A 1:1:1 mixture of OGG1, MSH2-MSH3 and an 80-mer oligonucleotide duplex containing one 8-oxo-G residue was resolved by gel-filtration column chromatography using a Sephacryl S-100 matrix (see Supplementary Method). Elution was monitored by absorbance at 260 nm. (**b**) Column fractions were resolved using SDS-PAGE and were probed using antibodies for OGG1 and MSH2-MSH3 (see Supplementary Method for antibody details). The OGG1 protein is a small protein (39 kD), while MSH2-MSH3 is 230 kD. They should not co-elute due to the size differences. As indicated by the elution profile and the immunoblotting analysis, no unbound OGG1 overlaps with MSH2-MSH3, indicating that the proteins do not associate off DNA. A product containing both OGG1 and MSH2-MSH3 bound to DNA would be larger than the MSH2-MSH3-DNA complex, and would be the fastest migrating species in the elution profile. However, as detected in the blots, no OGG1 or MSH2-MSH3 co-elutes with the fastest migrating species. Thus, the two proteins do not appear to interact, but are observed in one fraction where there is modest overlap of elution fractions.

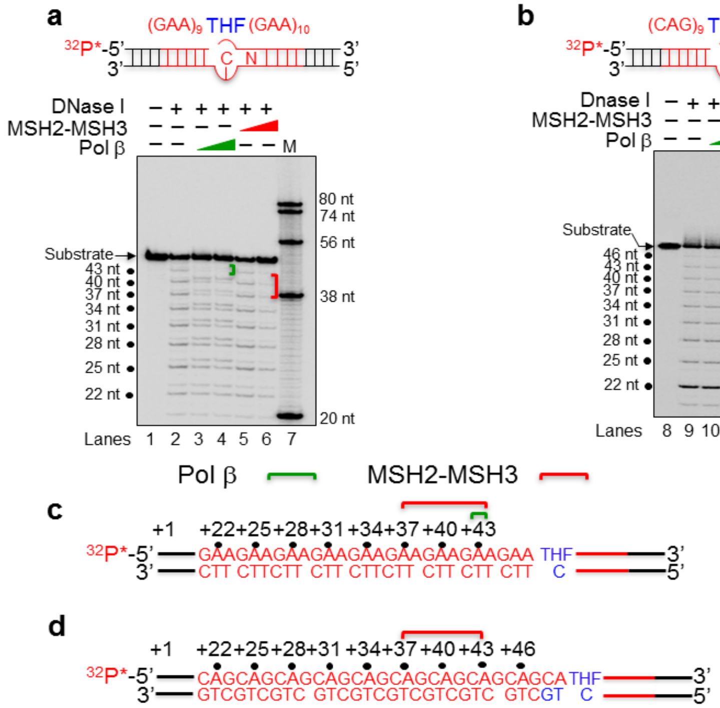

Supplementary Figure 3. There is no binding of MSH2-MSH3 and pol β on the damaged strand of (GAA)$_{20}$ and (CAG)$_{20}$ templates. (**a**) DNase I footprinting of pol β and MSH2-MSH3 on the (GAA)$_{20}$ substrate that is ^{32}P-labeled at the 5'-end. (top) A schematic diagram of the (GAA)$_{20}$ template depicting the position of the THF site (+53 in blue), and the ^{32}P-label on the 5'-end of the damaged strand. The binding sites of MSH2-MSH3 and pol β on TNR-containing BER intermediates were probed by DNase I digestion as described in the Methods. Pol β, MSH2-MSH3 and DNase I (0.008 U) were added as indicated above the gels; (+) is the presence of the component and (-) is the absence of the component. Lane 1 is the substrate only. Lane 2 is the unbound substrate digested with 0.008 U DNase I. The triangles indicate increasing concentrations of either pol β at 50 nM and 100 nM (in green) or MSH2-MSH3 at 100 nM and 200 nM (in red). Lane 7 represents synthesized markers (M), whose size is indicated to the right of the gel. No protection of MSH2-MSH3 and pol β occurs on the damaged strand. DNase I digestion sites are indicated to the left of the gel as black dots and the numbered positions of the cleavage product correspond to the numbering system in (**c**). (**b**) Same as **a** for the (CAG)$_{20}$ template. Experiments were done in triplicate.

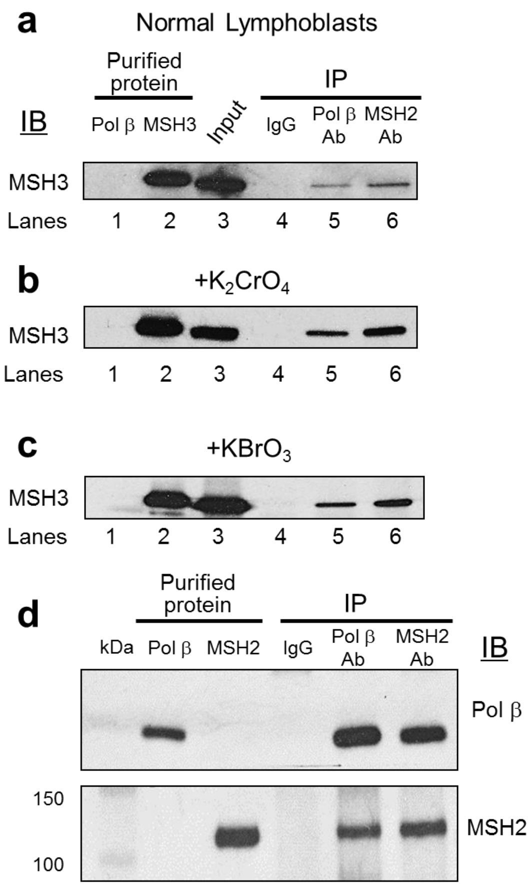

Supplementary Figure 4. MSH3 is present in the protein complexes that are immunoprecipitated with pol β or MSH2 antibody. The presence of MSH3 protein in the cell extracts derived from untreated normal lymphoblasts (GM02152) (**a**) or lymphoblasts treated with 0.5 mM K_2CrO_4 (**b**) or 10 mM $KBrO_3$ (**c**) for 2 hrs was detected with an anti-MSH3 antibody by immunoblotting (IB). Cell lysates were subjected to co-immunoprecipitation with pol β and MSH2 antibodies and immunoblotting for MSH3, as described in the Methods. Lanes 1 and 2 are the purified pol β or MSH3 proteins, as indicated. Lane 3 corresponds to cell lysates without treatment as an "Input" control. Lane 4 is the cell lysates immunoprecipitated with a rabbit IgG alone; Lanes 5 and 6 are cell lysates immunoprecipitated with an anti-pol β antibody and an anti-MSH2 antibody, respectively. IP is immunoprecipitation antibody; IB is immunoblotting antibody. Purified pol β and MSH3 proteins were employed as a molecular weight marker (lane 2). (**d**) Uncropped scans of the immunoblotting shown in **Fig. 3e**.

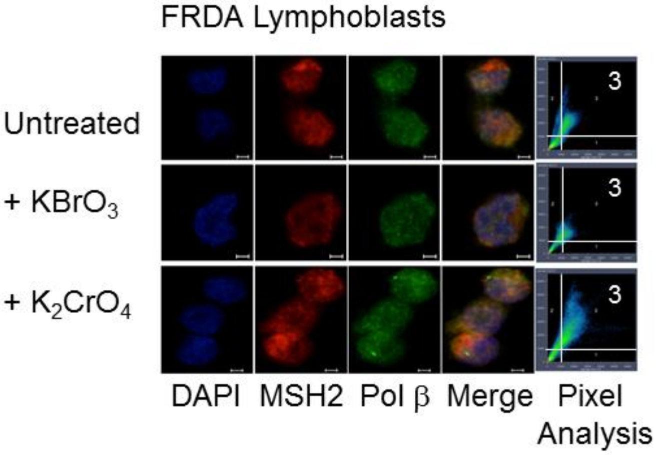

Supplementary Figure 5. MSH2 and pol β co-localize in FRDA lymphoblasts. Co-localization of MSH2 and pol β in lymphoblasts from a FRDA patient (GM02107) untreated or treated with oxidative DNA damaging-agents, potassium bromate or potassium chromate, as indicated. Representative images are illustrated. MSH2 (red), pol β (green), or merged images are indicated. Zeiss pixel analysis results are illustrated to the right side of each panel. The x- and y-axes represent red and green staining intensity, respectively. The white bars represent the quadrant thresholds below which pixels were deemed to contain only red or only green staining. The number 3 is the quadrant in which pixels contain both red and green intensities. DAPI (blue) is a stain for nuclear DNA; red is the anti-MSH2 staining; green is anti-pol β staining. Experiments were performed in triplicate.

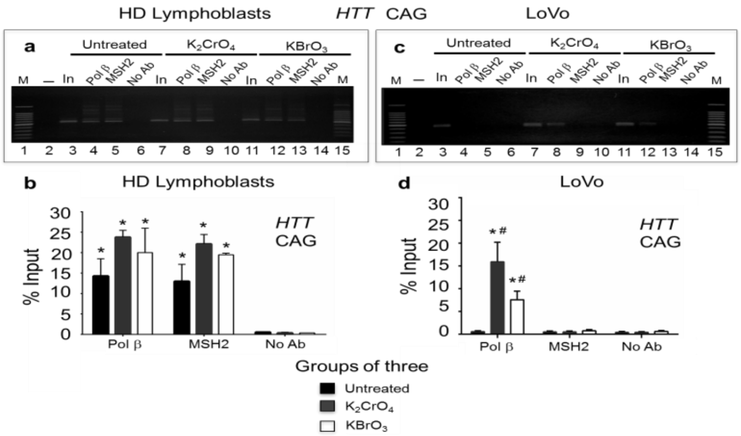

Supplementary Figure 6. Recruitment of MSH2 and pol β to CAG repeats of *Huntingtin* (*HTT*) gene in HD lymphoblasts. Recruitment of MSH2 and pol β to CAG repeats in *HTT* gene of lymphoblasts from a HD patient (GM13511) was detected by ChIP assay with an anti-MSH2 and anti-pol β antibody. The ChIP assay was conducted in LoVo (MSH2-deficient) cells as a control. (**a**) A representative agarose gel result showing recruitment of MSH2 and pol β to CAG repeats of *HTT* gene in untreated HD lymphoblasts and cells treated by chromate or bromate. (**b**) Quantification of **a**. (**c**) A representative agarose gel result showing recruitment of pol β to CAG repeats of *HTT* gene in LoVo cells after exposure to chromate or bromate. Untreated cells served as the control. Lanes 1 and 15 represent DNA size marker (M). Lane 2 represents the "Non-template" control. Lanes 3-6 represent "Input", Pol β-IP, MSH2-IP and No Ab control of untreated cells, respectively. Lanes 7-10 represent "Input", Pol β-IP, MSH2-IP and No Ab control of cells treated by chromate, respectively. Lanes 11-14 represent "Input", Pol β-IP, MSH2-IP and No Ab control of cells treated by chromate, respectively. (**d**) Quantification of **c**. Experiments were done in triplicate. The "Non-template" control, the no antibody control, and the input DNA are indicated for both treated and untreated cells. The bands were obtained using quantitative PCR and expressed as "% Input". All "% Input" were obtained from three independent experiments and expressed as mean ± s.d. Two-way ANOVA with Tukey's multiple comparison posttests was used to determine statistically significant differences. "*" denotes P < 0.05, compared to No Ab control, and "#" denotes P <0.05, compared with untreated cells.

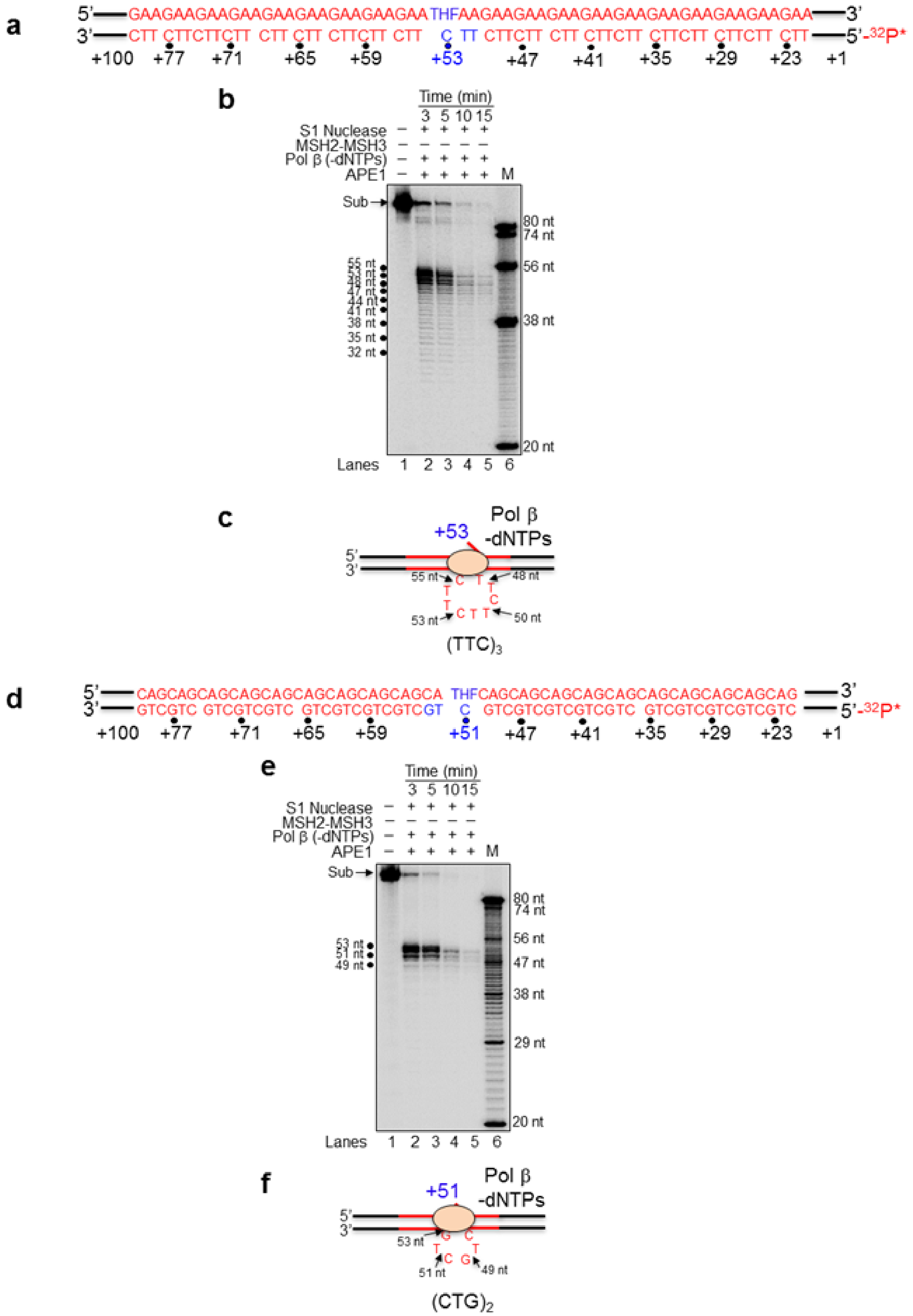

Supplementary Figure 7. Formation of a small loop on the template strand in the absence of active pol β synthesis. S1 nuclease cleavage on the template strand containing $(GAA)_{20}$ or $(CAG)_{20}$ in the presence of pol β protein in the absence of dNTPs was performed, and the

cleavage products were detected in the denaturing sequencing gel. (**a**) The $(GAA)_{20}$ repeat substrate containing an abasic site (the THF residue) was ^{32}P-labeled on the 5'-end of the template strand. (**b**) S1 nuclease cleavage on the template strand after APE1 5'-incision of the abasic site in the presence of pol β but without dNTPs. Lane 1 is the undigested substrate. The repair machinery present in each reaction is indicated above the gels; (+) is the presence of the component and (-) is the absence of the component. The sites of S1 cleavage are indicated on the left of the gel with major cut sites indicated by black dots, and correspond to the numbering system in **a**. The sizes of the synthesized DNA markers (M) are indicated to the right of the gel. The concentrations of the reagents in the reaction were as follows: substrate (100 nM), APE1 (10 nM), pol β (10 nM), and S1 nuclease (12 U). In all reactions, the substrates and BER components were pre-incubated with 10 nM APE1 prior to digestion with 12 U of S1 nuclease at the indicated time intervals at 37°C. (**c**) is the schematic summary of the S1 nuclease digestion using the numbering system in **a**. The black arrows and numbers indicate the positions of S1 cleavage. The orange ball is pol β. Red are the TNR repeats and black are the random sequences. (**d**-**f**) Same as in **a**-**c** for the $(CAG)_{20}$ substrate. Experiments were repeated in triplicate.

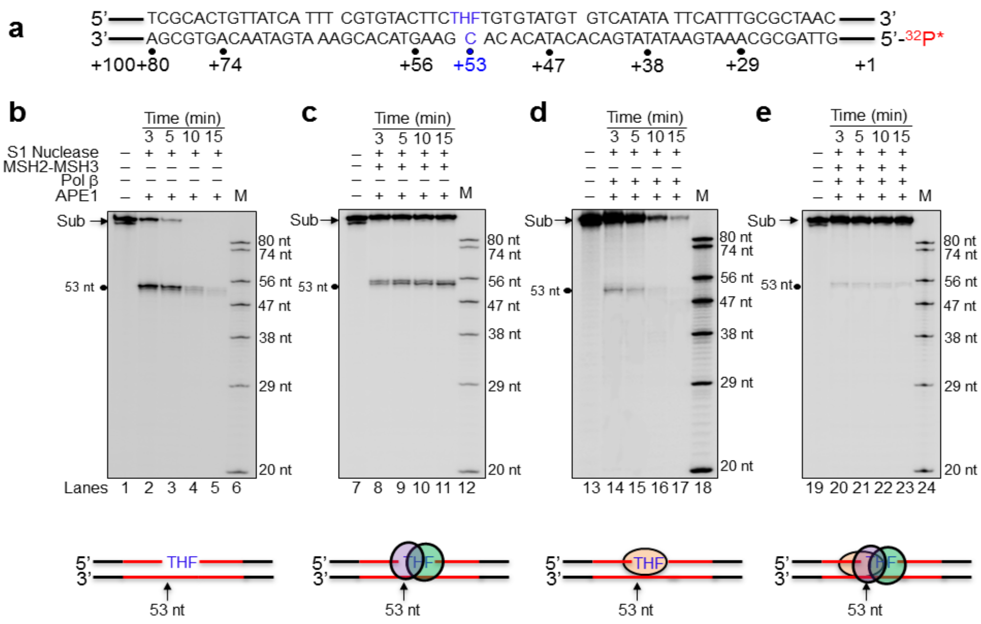

Supplementary Figure 8. No loop structure is detected in the context of random DNA sequences. The formation of secondary structures on the template strand of a random DNA sequence substrate containing an abasic lesion was probed by S1 nuclease digestion. (**a**) A schematic diagram of a random DNA sequence-containing BER substrate that was 5'-radiolabeled on the template strand. (**b**) The results of probing for template secondary structures formed on the random DNA sequence substrate after APE1 5'-incision of an abasic lesion. (**c**) The results of probing for template secondary structures in the random DNA sequence substrate in the presence of 100 nM MSH2-MSH3 alone. (**d**) The results of probing for template secondary structures in the random sequence substrate in the presence of pol β DNA synthesis. (**e**) The results of probing for template secondary structures in the random DNA sequence substrate in the presence of both 100 nM MSH2-MSH3 and pol β DNA synthesis at 10 nM. Lanes 1, 7, 13 and 19 represent the undigested substrate. Lanes 2−5, 8-11, 14-17 and 20-23 represent the digestion products generated at various time intervals. Lanes 6, 12, 18 and 24 represent synthesized size markers (M). An S1 nuclease cleavage site on the random sequence substrate was illustrated during BER in presence of pol β or MSH2-MSH3. Experiments were repeated at least three times.

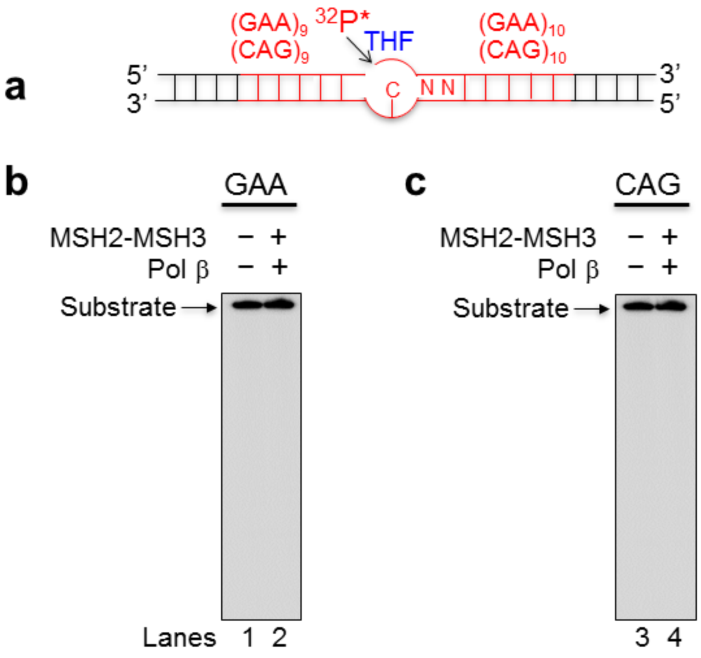

Supplementary Figure 9. MSH2-MSH3 cannot make flap cleavage in the presence of pol β DNA synthesis. (**a**) The schematic representation of the $(GAA)_{20}$ or $(CAG)_{20}$ substrates that were ^{32}P-labeled at the 5'-end of the downstream damaged strand. The $(GAA)_{20}$ (**b**) or $(CAG)_{20}$ (**c**) substrates (25 nM) were incubated with 100 nM MSH2-MSH3 and 10 nM pol β in the presence of dNTPs at 37°C for 15 min. The components present in reaction mixtures are indicated above the gels; (+) is the presence of the component, and (-) is the absence of the component. Lanes 1 and 3 correspond to the substrate only. Lanes 2 and 4 represent reaction mixtures that contained 100 nM MSH2-MSH3, 10 nM pol β and 50 μM dNTPs. Experiments were repeated in triplicate.

Supplementary Table 1 Oligonucleotides sequences

Oligonucleotides	nt	Sequence (5'-3')
**Damaged Strands**		
**DS1**	**99**	CGA GTC ATC TAG CAT CCG TA GAA GAA GAA GAA GAA GAA GAA GAA GAA **F**AA GAA GAA GAA GAA GAA GAA GAA GAA GAA GAA TA CGT AGA CTT ACT CAT TGC
**DS2**	**99**	CGA GTC ATC TAG CAT CCG TA CAG CAG CAG CAG CAG CAG CAG CAG CAG CA**F** CAG CAG CAG CAG CAG CAG CAG CAG CAG CAG TA CGT AGA CTT ACT CAT TGC
**DS3**	**99**	CGA GTC ATC TAG CAT CCG TA TCG CAC TGT TAT CAT TTC GTG TAC TTC **F**TG TGT ATG TGT CAT ATA TTC ATT TGC GCT AAC TA CGT AGA CTT ACT CAT TGC
**Upstream Strands**		
**U1**	**47**	CGA GTC ATC TAG CAT CCG TA GAA GAA GAA GAA GAA GAA GAA GAA GAA
**U2**	**49**	CGA GTC ATC TAG CAT CCG TA CAG CAG CAG CAG CAG CAG CAG CAG CAG CA
**Downstream Strands**		
**D1**	**52**	p**F**-AA GAA GAA GAA GAA GAA GAA GAA GAA GAA GAA TA CGT AGA CTT ACT CAT TGC
**D2**	**50**	p**F**-CAG CAG CAG CAG CAG CAG CAG CAG CAG CAG TA CGT AGA CTT ACT CAT TGC
**Template Strands**		
**T1**	**100**	GCA ATG AGT AAG TCT ACG TA TTC TTC TTC TTC TTC TTC TTC TTC TTC TTC TTC TTC TTC TTC TTC TTC TTC TTC TTC TTC TA CGG ATG CTA GAT GAC TCG
**T2**	**100**	GCA ATG AGT AAG TCT ACG TA CTG CTG CTG CTG CTG CTG CTG CTG CTG CTG CTG CTG CTG CTG CTG CTG CTG CTG CTG CTG TA CGG ATG CTA GAT GAC TCG
**T3**	**100**	GCA ATG AGT AAG TCT ACG TA GTT AGC GCA AAT GAA TAT ATG ACA CAT ACA CAT GAA GTA CAC GAA ATG ATA ACA GTG CGA TA CGG ATG CTA GAT GAC TCG

[a] The damaged base is in boldface. F, tetrahydofuran.

Supplementary Method

Gel-filtration column chromatography

A 1:1:1 mixture of OGG1, MSH2-MSH3, and an 80-mer oligonucleotide duplex containing one 8-oxo-G residue (in red) were resolved by gel-filtration column chromatography using a Sephacryl S-100 matrix. The CAG template was an 80 bp fragment containing 13 CAG repeats, a central 8-oxo-G lesion at position 17, and 20 bases of random sequence on each side. 5'-CAAGCACGTTGACTACCGTCCAGCAGCAGCAGCAGCAGCAGCAGCAGCAGCAGCAGCAG TTTGAGGCAGAGTCCGAACAC-3'**.** Column fractions along the elution profile were probed using antibodies for human OGG1 and MSH2-MSH3, and are shown below the elution curve. The fractions were heating in SDS loading buffer at 50°C for 10 min and subsequently resolved by SDS-PAGE and immunoblotted with mouse anti-human MSH2 antibodies (1:500; ab52266, Abcam, Cambridge, MA), or with primary antibodies for hOGG1 (1:1000; a kind gift from Tapas Hazra, University of Texas Medical Branch, Galveston, TX), followed by incubation with goat anti-rabbit IgG (1:10,000; ab6721, Abcam, Cambridge, MA) or rabbit anti-mouse IgG (1:7,000; ab6728, Abcam, Cambridge, MA). The blots were visualized by chemiluminescence using a Pierce ECL Western Blotting Substrate (#32106, Pierce-Thermo Scientific, Rockford, IL).

Supplementary Reference

1. Dianov, G. & Lindahl, T. Reconstitution of the DNA base excision-repair pathway. *Curr. Biol.* **4**, 1069-76 (1994).
